# 2,4,6-Trinitro­phenyl benzoate

**DOI:** 10.1107/S1600536812048362

**Published:** 2012-11-30

**Authors:** Rodolfo Moreno-Fuquen, Fabricio Mosquera, Alan R. Kennedy, Catriona A. Morrison, Regina H. De Almeida Santos

**Affiliations:** a Departamento de Química, Facultad de Ciencias, Universidad del Valle, Apartado 25360, Santiago de Cali, Colombia; b WestCHEM, Department of Pure and Applied Chemistry, University of Strathclyde, 295 Cathedral Street, Glasgow G1 1XL, Scotland; c Instituto de Química de São Carlos, IFSC, Universidade de São Paulo, USP, São Carlos, SP, Brazil

## Abstract

In the title mol­ecule, C_13_H_7_N_3_O_8_, the phenyl and benzene rings are rotated from the mean plane of the central ester group by 18.41 (9) and 81.80 (5)°, respectively. The dihedral angle between the rings is 80.12 (14)°. In the crystal, mol­ecules are linked by weak C—H⋯O inter­actions, forming helical chains along [010].

## Related literature

For theoretical and spectroscopic properties of nitro­phenyl esters, see: Ibrahim *et al.* (2011 ▶); Kirkien-Konasievicz & Maccoll (1964 ▶). For the structures of similar esters, see: Moreno-Fuquen *et al.* (2012*a* ▶,*b* ▶); Shibakami & Sekiya (1995 ▶); Gowda *et al.* (2007 ▶). For structural properties of nitro­phenyl compounds, see: Domenicano *et al.* (1990 ▶); Glidewell *et al.* (2005 ▶). For hydrogen-bonding information, see: Nardelli (1995 ▶). For a description of the Cambridge Structural Database, see: Allen (2002 ▶).
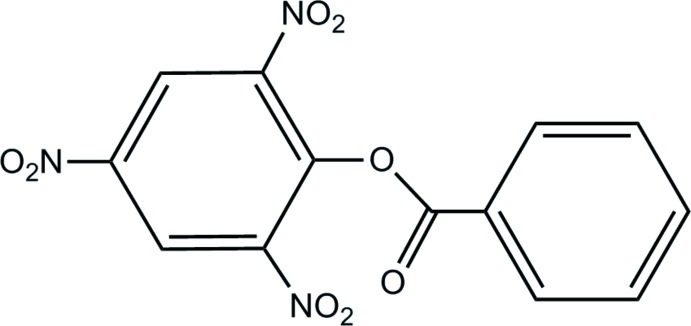


## Experimental

### 

#### Crystal data

C_13_H_7_N_3_O_8_
*M* *_r_* = 333.22
Orthorhombic, 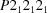

*a* = 7.5818 (3) Å
*b* = 8.3714 (2) Å
*c* = 21.0625 (10) Å
*V* = 1336.84 (9) Å^3^
*Z* = 4
Mo *K*α radiation
μ = 0.14 mm^−1^
*T* = 123 K
0.31 × 0.21 × 0.12 mm

#### Data collection

Oxford Diffraction Xcalibur E diffractometer
5179 measured reflections
2758 independent reflections
2563 reflections with *I* > 2σ(*I*)’
*R*_int_ = 0.019
Standard reflections: 0

#### Refinement

*R*[*F* ^2^ > 2σ(*F* ^2^)] = 0.032
*wR*(*F* ^2^) = 0.073
*S* = 1.05
2758 reflections
217 parameters
H-atom parameters constrained
Δρ_max_ = 0.17 e Å^−3^
Δρ_min_ = −0.21 e Å^−3^

### 

Data collection: *CrysAlis PRO* (Oxford Diffraction, 2010 ▶); cell refinement: *CrysAlis PRO*; data reduction: *CrysAlis PRO*; program(s) used to solve structure: *SHELXS97* (Sheldrick, 2008 ▶); program(s) used to refine structure: *SHELXL97* (Sheldrick, 2008 ▶); molecular graphics: *ORTEP-3 for Windows* (Farrugia, 2012 ▶) and *Mercury* (Macrae *et al.*, 2006 ▶); software used to prepare material for publication: *WinGX* (Farrugia, 2012 ▶).

## Supplementary Material

Crystal structure: contains datablock(s) I, global. DOI: 10.1107/S1600536812048362/lh5562sup1.cif

Structure factors: contains datablock(s) I. DOI: 10.1107/S1600536812048362/lh5562Isup2.hkl

Supplementary material file. DOI: 10.1107/S1600536812048362/lh5562Isup3.cml

Additional supplementary materials:  crystallographic information; 3D view; checkCIF report

## Figures and Tables

**Table 1:** Hydrogen-bond geometry (Å, °)

*D*—H⋯*A*	*D*—H	H⋯*A*	*D*⋯*A*	*D*—H⋯*A*
C3—H3⋯O8^i^	0.95	2.44	3.388 (2)	177

Symmetry code: (i) 
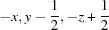
.

**Fig. 1.**
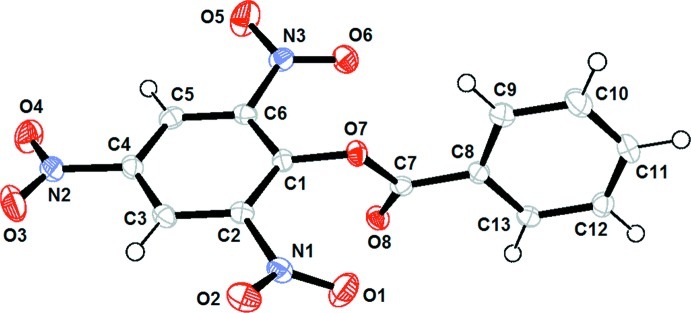
The molecular structure and atom numbering scheme for the title compound with displacement ellipsoids drawn at the 50% probability level. H atoms are shown as spheres of arbitrary radius.

**Fig. 2.**
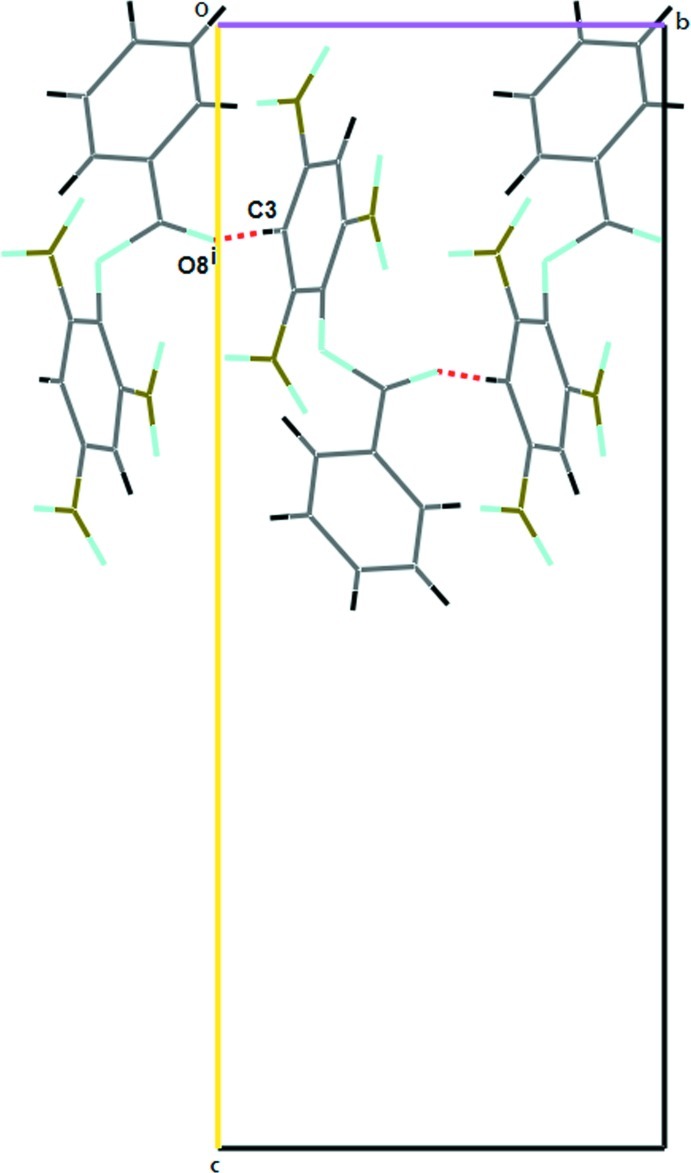
Part of the crystal structure of (I), showing the formation of helical chains along [010]. Symmetry code: (i) -x,+y-1/2,-z+1/2.

## supplementary crystallographic information

### Comment

In our research group, we have recently investigated the synthesis, crystalline properties and main structural features of nitro aryl benzoates, in particular, the derivatives of picric acid (trinitrophenol - TNP). These types of compounds have been synthetically obtained and studied at a spectroscopic and theoretical level (Ibrahim *et al.*, 2011) but there are no prior entries in Cambridge Structural Database (Version 5.33, Allen, 2002) of such related esters. Consequently, there is an absence of structural information about these esters. In order to fulfill this absence, we present the structure determination of the title compound, as part of a group of nitro aryl benzoates such as 2,4,6-trinitrophenyl-3-methylbenzoate (TNP3MeBA) and 2,4,6-trinitrophenyl-4-methylbenzoate (TNP4MeBA), the structures of which are already published (Moreno-Fuquen *et al.*, 2012*a*,*b*).

The molecular structure of (I) is shown in Fig. 1, with a numbering scheme similar to that for TNP3MeBA and TNP4MeBA in order to simplify structural comparisons. In (I) the bond lengths and bond angles agree closely with those for its homologous esters TNP3MeBA (Moreno-Fuquen *et al.*, 2012*a*) and TNP4MeBA (Moreno-Fuquen *et al.*, 2012*b*). However, if these structural parameters are strictly checked in other phenyl benzoates having no nitro substituent over the structure (Shibakami & Sekiya, 1995, Gowda *et al.*, 2007), it should be noticed that the phenolic C1—O7 bond length is significantly shortened [1.3695 (19) Å] and the benzoic C7—O7 bond length is significantly elongated [1.3876 (17) Å]. These changes in the bond parameters in (I) seem to be a feature of this kind of nitro phenyl benzoates , as they were found also in TNP3MeBA and TNP4MeBA, and are probably related with the presence of nitro substituents in the structure. Such effects have been well described by other authors (Kirkien-Konasievicz & Maccoll, 1964; Domenicano *et al.*, 1990; Ibrahim *et al.*, 2011) in a large varied number of other organic structures and can be rationalized in terms of inductive, resonance, reactivity and steric effects produced by nitro groups over aromatic rings. The benzene rings of (I) form a dihedral angle of 80.12 (14)°, compared with values of 87.48 (5)° and 69.02 (5)°, in TNP3MeBA and TNP4MeBA respectively. The central ester moiety forms an angle of 18.41 (9)° with the phenyl ring to which it is attached. This value is similar to the corresponding angle in the homologous ester TNP3MeBA 19.42 (7)°. The nitro groups form dihedral angles with the adjacent benzene ring of 29.55 (8)°, 13.94 (9)° and 8.84 (7)° for O1—N1—O2, O3—N2—O4 and O5—N3—O6, respectively. It was noted that for TNP4MeBA those same dihedral angles present almost identical values 30.57 (11)°, 14.75 (16)° and 7.37 (17)° and for TNP3MeBA the differences appear to increase 43.15 (10)°, 7.72 (14)° and 13.56 (18)°. The molecules are packed forming weak interactions C—H···O in one-dimensional helical chains which propagate along [010] (see Fig. 2). The C3 atom of the phenyl ring at (x,y,z) acts as a hydrogen-bond donor to carbonyl atom O8 at (-x,+y-1/2,-z+1/2) (see Table 1; Nardelli, 1995). This molecular interaction, involving the same atoms and given akin supramolecular behavior, is also found in TNP3MeBA and TNP4MeBA. For (I), the interaction is defined by distance D..A [3.388 (2) Å] and the angle D-H..A [176°], shown in Table 1, which compared with its analogous values in TNP4MeBA [3.4286 (19) Å, 175°] and TNP3MeBA [3.4276 (19) Å, 176°] reveal an extraordinary similarity at structural level among these group of compounds; very uncommon if it is taken into account that small changes in the molecular structures of a group of molecules usually lead to large changes in molecular aggregation of the structures (Glidewell *et al.*, 2005).

### Experimental

The reagents and solvents for the synthesis were obtained from the Aldrich Chemical Co., and were used without additional purification. The title molecule was synthesized using equimolar quantities of benzoyl chloride (0.235 g, 1.673 mmol) and picric acid (0.383g). The reagents were dissolved in acetonitrile and the solution was taken to reflux for about an hour. A pale yellow solid was obtained after leaving the solvent to evaporate. The solid was washed with distilled water and cold methanol to eliminate impurities. Crystals of good quality and suitable for single-crystal X-ray diffraction were grown from acetonitrile. IR spectra were recorded on a FT—IR SHIMADZU IR-Affinity-1 spectrophotometer. Pale Yellow crystals; yield 72%; m.p 435 K. IR (KBr) 3082.38 cm^-1^, 3025.48 cm^-1^ (aromatic C—H); 1759.15 cm^-1^ (ester C=O); 1618.34 cm^-1^, 1611.59 cm^-1^ (C=C); 1544.08 cm^-1^, 1342.51 cm^-1^ (–NO2); 1224.85 cm^-1^ (C(=O)—O).

### Refinement

All H-atoms were positioned at geometrically idealized positions with C—H distance of 0.93 Å and U_iso_(H) = 1.2 times U_eq_ of the C-atoms to which they were bonded.

### Crystal data

**Table:**

C_13_H_7_N_3_O_8_	*D*_x_ = 1.656 Mg m^−^^3^
*M**_r_* = 333.22	Melting point: 435(1) K
Orthorhombic, *P*2_1_2_1_2_1_	Mo *K*α radiation, λ = 0.71073 Å
Hall symbol: P 2ac 2ab	Cell parameters from 5179 reflections
*a* = 7.5818 (3) Å	θ = 2.9–27.0°
*b* = 8.3714 (2) Å	µ = 0.14 mm^−^^1^
*c* = 21.0625 (10) Å	*T* = 123 K
*V* = 1336.84 (9) Å^3^	Block, pale-yellow
*Z* = 4	0.31 × 0.21 × 0.12 mm
*F*(000) = 680	

### Data collection

**Table:**

Oxford Diffraction Xcalibur E diffractometer	2563 reflections with *I* > 2σ(*I*)'
Radiation source: fine-focus sealed tube	*R*_int_ = 0.019
Graphite monochromator	θ_max_ = 27.0°, θ_min_ = 2.9°
ω scans	*h* = −9→8
5179 measured reflections	*k* = −10→10
2758 independent reflections	*l* = −26→23

### Refinement

**Table:**

Refinement on *F*^2^	Primary atom site location: structure-invariant direct methods
Least-squares matrix: full	Secondary atom site location: difference Fourier map
*R*[*F*^2^ > 2σ(*F*^2^)] = 0.032	Hydrogen site location: inferred from neighbouring sites
*wR*(*F*^2^) = 0.073	H-atom parameters constrained
*S* = 1.05	*w* = 1/[σ^2^(*F*_o_^2^) + (0.0354*P*)^2^ + 0.1599*P*] where *P* = (*F*_o_^2^ + 2*F*_c_^2^)/3
2758 reflections	(Δ/σ)_max_ < 0.001
217 parameters	Δρ_max_ = 0.17 e Å^−^^3^
0 restraints	Δρ_min_ = −0.21 e Å^−^^3^

### Special details

**Table:**

Geometry. All esds (except the esd in the dihedral angle between two l.s. planes) are estimated using the full covariance matrix. The cell esds are taken into account individually in the estimation of esds in distances, angles and torsion angles; correlations between esds in cell parameters are only used when they are defined by crystal symmetry. An approximate (isotropic) treatment of cell esds is used for estimating esds involving l.s. planes.
Refinement. Refinement of *F*^2^ against ALL reflections. The weighted *R*-factor *wR* and goodness of fit *S* are based on *F*^2^, conventional *R*-factors *R* are based on *F*, with *F* set to zero for negative *F*^2^. The threshold expression of *F*^2^ > σ(*F*^2^) is used only for calculating *R*-factors(gt) *etc*. and is not relevant to the choice of reflections for refinement. *R*-factors based on *F*^2^ are statistically about twice as large as those based on *F*, and *R*- factors based on ALL data will be even larger.

### Fractional atomic coordinates and isotropic or equivalent isotropic displacement parameters (Å^2^)

**Table:**

	*x*	*y*	*z*	*U*_iso_*/*U*_eq_	
O1	0.11331 (19)	0.69680 (17)	0.15456 (6)	0.0370 (3)	
O2	−0.03793 (16)	0.51649 (14)	0.20396 (6)	0.0265 (3)	
O3	−0.10783 (19)	0.58818 (19)	0.43018 (7)	0.0423 (4)	
O4	0.07057 (19)	0.74602 (16)	0.48041 (6)	0.0344 (3)	
O5	0.6229 (2)	0.8632 (2)	0.38383 (7)	0.0479 (4)	
O6	0.65305 (16)	0.87515 (14)	0.28252 (6)	0.0275 (3)	
O7	0.42129 (15)	0.73147 (12)	0.21060 (5)	0.0182 (2)	
O8	0.35960 (15)	0.99125 (13)	0.19172 (5)	0.0205 (3)	
N1	0.07053 (19)	0.62411 (16)	0.20235 (6)	0.0207 (3)	
N2	0.01984 (19)	0.67714 (17)	0.43257 (7)	0.0246 (3)	
N3	0.56820 (19)	0.84505 (16)	0.33002 (7)	0.0219 (3)	
C1	0.3206 (2)	0.73479 (17)	0.26457 (8)	0.0159 (3)	
C2	0.1511 (2)	0.67083 (17)	0.26317 (8)	0.0170 (3)	
C3	0.0513 (2)	0.64857 (17)	0.31728 (8)	0.0186 (3)	
H3	−0.0619	0.6002	0.3155	0.022*	
C4	0.1230 (2)	0.69963 (18)	0.37407 (8)	0.0192 (3)	
C5	0.2881 (2)	0.76870 (18)	0.37826 (8)	0.0194 (3)	
H5	0.3328	0.8051	0.4178	0.023*	
C6	0.3864 (2)	0.78346 (17)	0.32340 (8)	0.0177 (3)	
C7	0.4341 (2)	0.87175 (17)	0.17581 (7)	0.0168 (3)	
C8	0.5485 (2)	0.84810 (18)	0.12011 (7)	0.0170 (3)	
C9	0.6668 (2)	0.7207 (2)	0.11679 (8)	0.0237 (4)	
H9	0.6743	0.6461	0.1506	0.028*	
C10	0.7730 (2)	0.7040 (2)	0.06380 (9)	0.0309 (4)	
H10	0.8530	0.6170	0.0610	0.037*	
C11	0.7630 (2)	0.8137 (2)	0.01489 (9)	0.0268 (4)	
H11	0.8373	0.8020	−0.0211	0.032*	
C12	0.6461 (2)	0.94018 (19)	0.01778 (8)	0.0206 (3)	
H12	0.6403	1.0150	−0.0161	0.025*	
C13	0.5369 (2)	0.95746 (18)	0.07042 (7)	0.0178 (3)	
H13	0.4550	1.0432	0.0725	0.021*	

### Atomic displacement parameters (Å^2^)

**Table:**

	*U*^11^	*U*^22^	*U*^33^	*U*^12^	*U*^13^	*U*^23^
O1	0.0388 (8)	0.0502 (8)	0.0220 (7)	−0.0188 (7)	−0.0061 (6)	0.0083 (6)
O2	0.0218 (6)	0.0228 (5)	0.0347 (7)	−0.0062 (5)	−0.0041 (6)	−0.0043 (5)
O3	0.0269 (7)	0.0656 (9)	0.0345 (8)	−0.0114 (8)	0.0099 (6)	0.0052 (7)
O4	0.0417 (8)	0.0419 (7)	0.0197 (6)	0.0053 (7)	0.0050 (6)	−0.0049 (6)
O5	0.0378 (8)	0.0807 (11)	0.0254 (8)	−0.0282 (9)	−0.0114 (7)	0.0050 (7)
O6	0.0211 (6)	0.0316 (6)	0.0299 (7)	−0.0083 (6)	0.0011 (6)	0.0042 (6)
O7	0.0190 (6)	0.0165 (5)	0.0190 (6)	−0.0007 (5)	0.0043 (5)	0.0018 (4)
O8	0.0201 (6)	0.0188 (5)	0.0227 (6)	0.0028 (5)	0.0031 (5)	0.0011 (5)
N1	0.0169 (7)	0.0223 (6)	0.0230 (7)	−0.0011 (6)	0.0003 (6)	−0.0027 (6)
N2	0.0214 (7)	0.0292 (7)	0.0233 (8)	0.0068 (7)	0.0044 (7)	0.0035 (6)
N3	0.0204 (7)	0.0217 (6)	0.0236 (8)	−0.0044 (6)	−0.0037 (6)	0.0032 (6)
C1	0.0179 (8)	0.0120 (6)	0.0178 (8)	0.0017 (6)	0.0023 (6)	0.0019 (6)
C2	0.0171 (8)	0.0143 (6)	0.0195 (8)	0.0010 (7)	−0.0012 (7)	−0.0010 (6)
C3	0.0154 (7)	0.0162 (7)	0.0241 (8)	0.0011 (7)	0.0022 (7)	0.0015 (6)
C4	0.0189 (8)	0.0177 (7)	0.0211 (9)	0.0037 (7)	0.0040 (7)	0.0026 (6)
C5	0.0231 (8)	0.0160 (7)	0.0190 (8)	0.0027 (7)	−0.0027 (7)	−0.0001 (6)
C6	0.0151 (8)	0.0147 (6)	0.0232 (8)	−0.0011 (6)	−0.0026 (7)	0.0020 (6)
C7	0.0138 (7)	0.0176 (7)	0.0188 (8)	−0.0026 (7)	−0.0030 (6)	0.0016 (6)
C8	0.0145 (7)	0.0188 (7)	0.0176 (8)	−0.0036 (7)	−0.0002 (7)	0.0005 (6)
C9	0.0211 (8)	0.0263 (8)	0.0237 (9)	0.0051 (8)	0.0011 (7)	0.0033 (7)
C10	0.0266 (9)	0.0330 (9)	0.0329 (10)	0.0116 (9)	0.0076 (9)	0.0032 (8)
C11	0.0220 (9)	0.0338 (9)	0.0244 (9)	0.0038 (8)	0.0078 (8)	−0.0007 (7)
C12	0.0205 (9)	0.0236 (7)	0.0176 (8)	−0.0039 (7)	0.0006 (7)	0.0008 (6)
C13	0.0153 (8)	0.0177 (7)	0.0203 (8)	−0.0007 (7)	−0.0014 (7)	−0.0011 (6)

### Geometric parameters (Å, º)

**Table:**

O1—N1	1.2201 (18)	C3—H3	0.9500
O2—N1	1.2203 (17)	C4—C5	1.382 (2)
O3—N2	1.222 (2)	C5—C6	1.381 (2)
O4—N2	1.2231 (18)	C5—H5	0.9500
O5—N3	1.2166 (19)	C7—C8	1.473 (2)
O6—N3	1.2158 (17)	C8—C13	1.393 (2)
O7—C1	1.3695 (19)	C8—C9	1.395 (2)
O7—C7	1.3876 (17)	C9—C10	1.383 (2)
O8—C7	1.1965 (18)	C9—H9	0.9500
N1—C2	1.472 (2)	C10—C11	1.382 (3)
N2—C4	1.471 (2)	C10—H10	0.9500
N3—C6	1.478 (2)	C11—C12	1.382 (2)
C1—C2	1.393 (2)	C11—H11	0.9500
C1—C6	1.396 (2)	C12—C13	1.391 (2)
C2—C3	1.381 (2)	C12—H12	0.9500
C3—C4	1.382 (2)	C13—H13	0.9500
			
C1—O7—C7	117.39 (11)	C5—C6—C1	121.58 (14)
O1—N1—O2	124.77 (14)	C5—C6—N3	117.12 (14)
O1—N1—C2	118.37 (13)	C1—C6—N3	121.22 (15)
O2—N1—C2	116.84 (13)	O8—C7—O7	121.79 (14)
O3—N2—O4	124.76 (16)	O8—C7—C8	127.85 (14)
O3—N2—C4	117.67 (15)	O7—C7—C8	110.37 (12)
O4—N2—C4	117.55 (14)	C13—C8—C9	120.35 (15)
O6—N3—O5	124.07 (15)	C13—C8—C7	118.21 (14)
O6—N3—C6	119.22 (14)	C9—C8—C7	121.43 (14)
O5—N3—C6	116.70 (14)	C10—C9—C8	119.47 (16)
O7—C1—C2	119.27 (14)	C10—C9—H9	120.3
O7—C1—C6	122.92 (14)	C8—C9—H9	120.3
C2—C1—C6	117.42 (14)	C11—C10—C9	120.15 (16)
C3—C2—C1	122.70 (15)	C11—C10—H10	119.9
C3—C2—N1	117.07 (14)	C9—C10—H10	119.9
C1—C2—N1	120.24 (14)	C10—C11—C12	120.75 (16)
C2—C3—C4	117.22 (15)	C10—C11—H11	119.6
C2—C3—H3	121.4	C12—C11—H11	119.6
C4—C3—H3	121.4	C11—C12—C13	119.75 (16)
C3—C4—C5	122.76 (16)	C11—C12—H12	120.1
C3—C4—N2	118.45 (14)	C13—C12—H12	120.1
C5—C4—N2	118.79 (15)	C12—C13—C8	119.52 (15)
C6—C5—C4	118.24 (15)	C12—C13—H13	120.2
C6—C5—H5	120.9	C8—C13—H13	120.2
C4—C5—H5	120.9		
			
C7—O7—C1—C2	101.44 (16)	O7—C1—C6—C5	−172.62 (13)
C7—O7—C1—C6	−85.90 (18)	C2—C1—C6—C5	0.2 (2)
O7—C1—C2—C3	170.39 (13)	O7—C1—C6—N3	4.1 (2)
C6—C1—C2—C3	−2.7 (2)	C2—C1—C6—N3	176.85 (13)
O7—C1—C2—N1	−9.8 (2)	O6—N3—C6—C5	−172.63 (14)
C6—C1—C2—N1	177.14 (13)	O5—N3—C6—C5	7.0 (2)
O1—N1—C2—C3	149.89 (15)	O6—N3—C6—C1	10.5 (2)
O2—N1—C2—C3	−28.6 (2)	O5—N3—C6—C1	−169.84 (16)
O1—N1—C2—C1	−29.9 (2)	C1—O7—C7—O8	−0.5 (2)
O2—N1—C2—C1	151.56 (14)	C1—O7—C7—C8	179.19 (13)
C1—C2—C3—C4	3.0 (2)	O8—C7—C8—C13	−18.8 (3)
N1—C2—C3—C4	−176.85 (13)	O7—C7—C8—C13	161.59 (14)
C2—C3—C4—C5	−0.8 (2)	O8—C7—C8—C9	160.83 (17)
C2—C3—C4—N2	−179.85 (13)	O7—C7—C8—C9	−18.8 (2)
O3—N2—C4—C3	13.6 (2)	C13—C8—C9—C10	0.2 (3)
O4—N2—C4—C3	−168.01 (14)	C7—C8—C9—C10	−179.38 (16)
O3—N2—C4—C5	−165.48 (15)	C8—C9—C10—C11	0.6 (3)
O4—N2—C4—C5	12.9 (2)	C9—C10—C11—C12	−0.7 (3)
C3—C4—C5—C6	−1.6 (2)	C10—C11—C12—C13	−0.1 (3)
N2—C4—C5—C6	177.51 (14)	C11—C12—C13—C8	0.9 (2)
C4—C5—C6—C1	1.9 (2)	C9—C8—C13—C12	−1.0 (2)
C4—C5—C6—N3	−174.95 (13)	C7—C8—C13—C12	178.63 (15)

### Hydrogen-bond geometry (Å, º)

**Table:**

*D*—H···*A*	*D*—H	H···*A*	*D*···*A*	*D*—H···*A*
C3—H3···O8^i^	0.95	2.44	3.388 (2)	177

Symmetry code: (i) −*x*, *y*−1/2, −*z*+1/2.
